# A Hydrothermal Synthesis of Fe_3_O_4_@C Hybrid Nanoparticle and Magnetic Adsorptive Performance to Remove Heavy Metal Ions in Aqueous Solution

**DOI:** 10.1186/s11671-018-2580-8

**Published:** 2018-06-13

**Authors:** Siping Ji, Changlin Miao, Hui Liu, Lili Feng, Xiangjun Yang, Hong Guo

**Affiliations:** 1 School of Chemistry Science and Engineering, Yunnan University, No. 2, CuiHu North Road, Kunming, 650091 China; 2 Joint Research Centre for International Cross-border Ethnic Regions Biomass Clean Utilization in Yunnan, Yunnan University of Nationalities, Kunming, 650500 China

**Keywords:** Composites, Nanomaterials, Heavy metal ions, Adsorption

## Abstract

Advanced core-shelled material with a high specific area has been considered as an effective material to remove heavy metal from aqueous solutions. A core-shelled Fe_3_O_4_@C hybrid nanoparticle aggregates with environmental-friendly channel in the study. Moreover, the higher exposure of adsorption sites can be achieved for the special configuration that higher Brunauer-Emmet-Teller (BET) surface area reaches up to 238.18 m^2^ g^−1^. Thus, a more efficiently heavy metal ion removal is obtained, Pb (II), Cd (II), Cu (II), and Cr (VI) up to 100, 99.2, 96.6, and 94.8%, respectively. In addition, the products are easy to be separated from the aqueous solutions after adsorption, due to the relative large submicrometer size and the enhanced external magnetic fields introduced by the iron-based cores. We provide an ideal mode to remove heavy metal ions using core-shelled Fe_3_O_4_@C under the water treatment condition. A new approach is clarified that core-shell nano/micro-functional materials can be synthesized well on large scales which are used in many fields such as environmental remediation, catalyst, and energy.

## Background

With the voice of environmental protection is constant and surging, there is increasing attention to its toxic effect that refers to human health and environmental pollution by heavy metal contamination [1–3]. Removing heavy metal elements from industrial wastewater prior to discharge becomes crucial [4]. To date, ion exchange, coagulation precipitation, and series of traditional technologies were applied to remove heavy metals from wastewater during the past decades [5, 6]. The realization that conventional techniques are significantly created benefits for human, and it also exposed drawbacks characterized both from management and technical sides, which are expensive operation cost, additional toxic sludge generation, and incomplete metal removal [7–9]. On the other hand, controlling particle size, the morphology of adsorbent materials, has proved to be one of the efficient and innovating solutions to conquer those kinds of problems. Hollow spheres, nanowires, and nanotubes have got a better adsorption performance to contribute to the heavy metal removal in References [10–13]. Further, researches are focused on the new material that holds core-shell structure with core-void-shell feature. It has been proved for its advantages compared with the same size of solid counterparts, such as the validity of that in the changes of surface areas, refractive index, lower density, and accommodate volume, which lead to a great contribution both from the aspects of properties and functions [14, 15]. Thus, this unique structure with tunable shape, composition, and interior architecture is an exciting direction to pursue environmental remediation.

Many literatures are engaged about core-shell structure materials. Guo [16] had made cage-bell Ag@TiO_2_ materials, and the study expressed better photocatalytic and electrochemical properties. Liu [17] prepared core-shell Fe_3_O_4_ polydopamine nanoparticles that exhibited nice potential in the field of medicine support, catalyst carrier, and carbon adsorbent. To our best knowledge, the assistance of removable or sacrificial templates (e.g., polymer silica [18], spheres [19], carbon [20], and ionic liquids [21]) of the desirable shells are important in general synthesis of core-shell hybrid nanoparticle structures. However, the most available core-shell structure materials are synthesized by multi-template processes that mainly focus on the relatively simple configurations, like one composition in single-shell particles. Moreover, there are still lacking in heavy metal ion removal method with a general approach accompany with further strengthen the prepare feasibility of the advanced materials with core-shell structures, including time and cost in the construction process of complex nanostructures that were limited by synthesis templates and multi-template routes, which have become a desire both from technical and eco-benefit aspect.

The synthesis of the magnetic functional nanocomposite is an effective and handy way to solve the separation between the adsorbent and solution for expanding the magnetic separation [22]. Increasingly covalently immobilized polymer, novel molecule, and inorganic material are put into the surface of magnetic nanoparticle in this process; they are useful technique routes for toxic heavy metal ion wastewater treatment as well [23]. For instance, novel synthesized chitosan-modified magnetic nanocomposites [24] and monodisperse Fe_3_O_4_@silica core-shell structure composite magnetic nanoparticle core-shell microspheres [25] are reported. Despite these magnetic nanocomposites achieved easily separated from solution through the adsorption process based on the external magnetic, it still needs to be further considered the special conditions such as the applicability of strong acid wastewater.

There are lots of studies about carbon-based nanostructured materials recently. Wildgoose [26] presented these kinds of materials hold obvious advantages in terms of cost, alkali corrosion resistance, specific surface area, and adsorption capacity. Uchida [27] pointed out that the carboxylic functional groups can easily generate on the surface of carbon then further enhance the adsorption capacity of heavy metal ions. However, the fatal flaw of the difficulty of removing them from a solution that caused by the small size of carbon particles limited its application in heavy metal wastewater treatment. Considering carbon-coated magnetic nanoparticle is a toward media in wastewater treatment, it showed advanced impacts on adsorption capacity and separation property in the external magnetic field. Much more attention has increased [28–35]. Zhang [36] prepared magnetic hollow carbon nanospheres forward used in chromium ion removal. To remove heavy metal, Wang groups [37] reported a case study of Fe nanoparticle materials. These previous studies indicated that the corresponding future work must refer to highly efficient heavy metal ion adsorbents with easy separation and large adsorption capacity. Meantime, it should be pointed out that there are rare studies on core-shelled Fe_3_O_4_@C hybrid nanoparticle aggregates up to now.

In this study, we prepared carbon microspheres with magnetic cores. Also, a concise strategy was proposed to synthesize core-shelled Fe_3_O_4_@C hybrid nanoparticle aggregates, which is an advanced material for heavy metal ion removal with the strength purity, surface areas, and adsorption capacity. Compared with traditional production technology of Fe_3_O_4_ materials, the benefits are obvious. It not only expressed a larger surface area and steady configuration, but also the removal template which is not affected by product morphology. Our research provides a higher degree of the active sites [38, 39]. The adsorbent could be easily separated when the external magnetic fields are introduced, which are caused by the iron-based nanoparticles [40, 41]. Therefore, the obtained core-shelled Fe_3_O_4_@C hybrid nanoparticle aggregates show superior adsorption capacity for heavy metal ions with the route eco-friendly, mass-production, and cost benefits.

## Experimental

### Materials and Synthesis

#### Synthesis of Core-Shelled Fe_3_O_4_ Hybrid Nanoparticle Aggregates

**Scheme 1:**
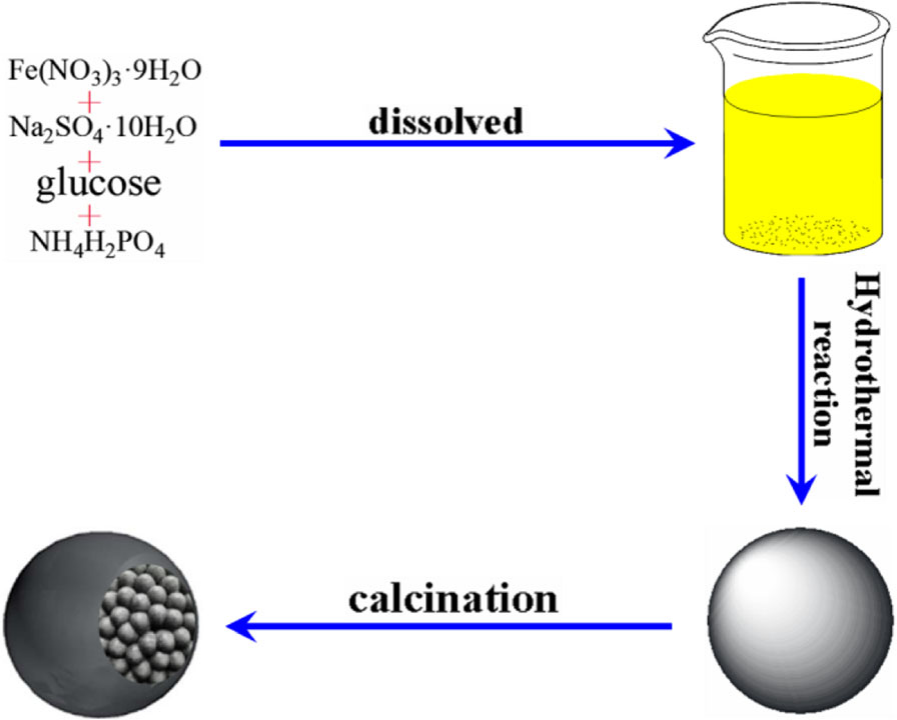
Synthesis route of the core-shelled Fe_3_O_4_@C hybrid nanoparticle

In typical synthesis steps, 0.72 g of Fe(NO_3_)_3_·9H_2_O, 0.0086 g of NH_4_H_2_PO_4_, 0.008 g of Na_2_SO_4_·10H_2_O, and 3 g of glucose were dissolved in distilled water, respectively, mixed all together in 90 mL volume, added distilled water to the mixture, and continuously mixed for 10 min with a basic magnetic stirrer. Then, the admixture shifted into 100 mL of Teflon-lined stainless-steel autoclave for 180 °C, 48 h. After the reaction is completed and cooled to room temperature naturally, the black foot was acquired and washed several times with deionized water and absolute ethanol, then desiccated the black foot at 65 °C the whole night under the condition of vacuum, and lastly, obtained precursors by calcined at 450 °C with the rate of 3 °C min^−1^ then retained heating with a sequential carbon monoxide/argon gas flow (4 h) and cooled down to the ambient temperature. The Fe_3_O_4_@C sample was obtained. As described in Scheme 1 the hydrolysis of Fe^3+^ leads to Fe oxide layer in nanometer accuracy. Meanwhile, sucrose is carbonized. After that, microspheres of Fe-C-O composite material will create in situ during the bath heating reaction through calcination sections and then gain core-shelled Fe_3_O_4_@C hybrid nanoparticle.

#### Characterization

The phase composition of prepared material was analyzed by X-ray diffraction (XRD), which was taken at 2*θ* = 20°–90° by Rigaku D/max-A diffractometer with Co Kα radiation. FTIR (Fourier transform infrared spectroscopy, Thermo Nicolet AVATARFTIR 360) was carried out to record the samples’ FTIR characters within the ranged 400–4000 cm^−1^ as well. AMRAY 1000B SEM (Scanning electron microscope), HR-TEM (High-resolution transmission electron microscope, JEOL JEM-2100) (200 Kv), and selected area electron diffraction (SAED) were implemented to describe the sample’s morphology, the microstructure feature, and the lattice structure. In addition, Micromeritics Tristar apparatus at 350.15 °C was taken to measure the processes of nitrogen adsorption and desorption; Brunauer-Emmet-Teller (BET) was used to discuss the specific surface areas; atomic absorption spectroscopy (AAS) quantitative analysis will be implemented by Hitachi Z2000 spectrophotometer, which fitted with hollow cathode lamps and acetylene-air flame. The magnetic performance of prepared material was measured by vibrating-sample magnetometer (VSM).

#### Heavy Metal Ion Removal Experiments

At room temperature, the conduct of a series of experiments was considered to remove heavy metal ions. First of all, Pb (II), Cd (II), Cu (II), and Cr (VI) are added in four closed containers; after that, 0.1 M HCl and 0.1 M NH_3_•H_2_O are used to adjust the pH to 3, and then, the adsorption solutions with the final volume of 50 mL and concentration of 10 mg L^−1^ were obtained. Subsequently, under a continuous stirring condition, 20 mg as-prepared Fe_3_O_4_@C sample was added to the above solutions. During the adsorb reaction process, almost 1.0 mL of each aforementioned solution was leached over the various periods (0, 0.5, 1, 1.5, 2, 4, 6, 10, and 24 h, respectively) by means of a pin tube utilization which equipped with membrane filter, attenuated at last to be applied to AAS measure.

## Result and Discussion

### Physicochemical Characteristics of Core-Shelled Fe_3_O_4_@C Nanosphere

**Fig. 1:**
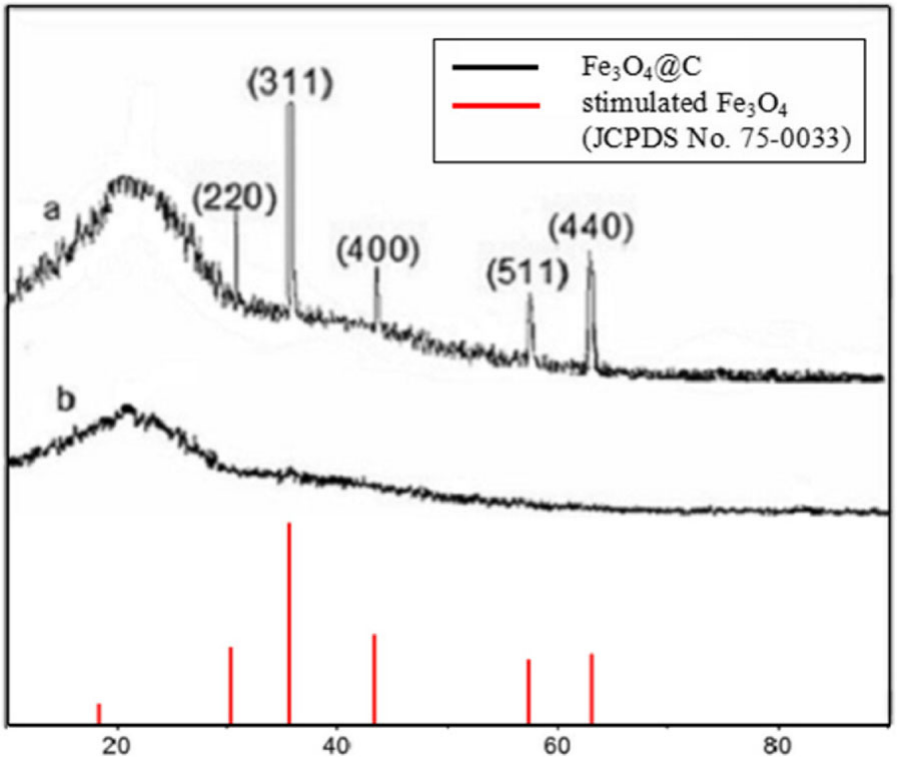
XRD patterns of core-shelled Fe_3_O_4_@C hybrid nanoparticle aggregates and its precursor (a—Fe_3_O_4_@C hybrid nanoparticle aggregates; b—the precursor)

The XRD patterns of the synthesized core-shelled Fe_3_O_4_@C hybrid nanoparticle aggregates and its precursor were shown in Fig. 1, which presented obviously that the production process is of higher crystallinity than the precursor through calcinations at 450 °C. The prepared samples displayed composite materials corresponding to face-centered cubic (f c c) Fe_3_O_4_ (JCPDS (Joint Committee on Powder Diffraction Standards) card no. 75-0033). The diffraction peak at 21.5° as shown in the patterns should be assigned to the (002) plane of graphitic carbon which can also be directly found by SEM and TEM.

**Fig. 2:**
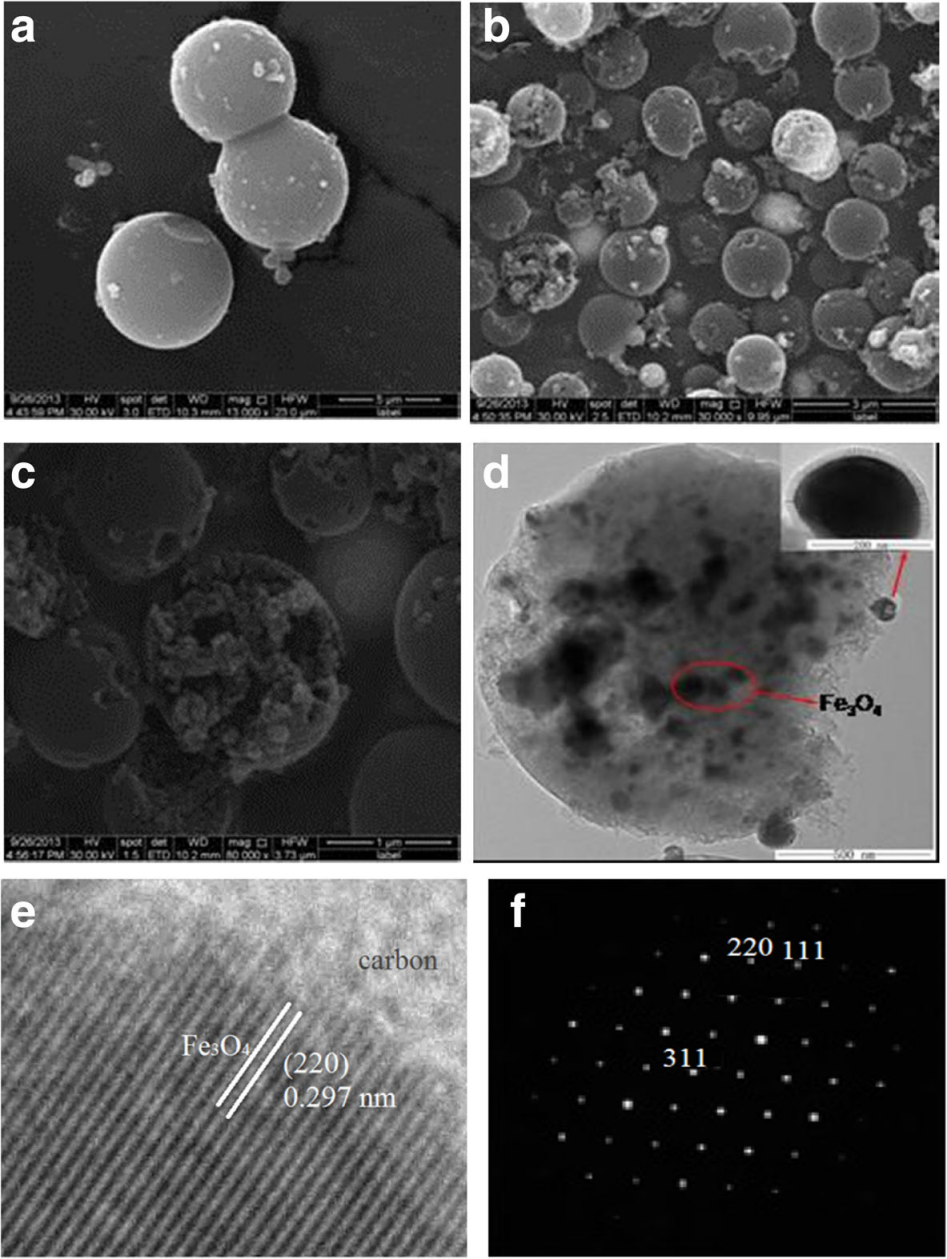
SEM image of prepared precursor (**a**). SEM images (**b**, **c**), TEM image (**d**), HRTEM micrograph (**e**), and selected area electron diffraction (**f**) of as-synthesized core-shelled Fe_3_O_4_@C hybrid nanoparticle aggregates yielded by calcinations at 450 °C

SEM images of the precursor and core-shelled Fe_3_O_4_@C hybrid nanoparticle synthesis produced via calcination at 450 °C at different magnifications were displayed in Fig. 2a–c. Obviously, apart from a little minish in size, the synthesized Fe_3_O_4_@C retained the morphology of the precursor prepared that are microspheres ca. with the size of 700 nm uniformly. Figure 2b, c clearly described the hybrid core-shelled structure of the Fe_3_O_4_@C; it can be evidenced by the mesospheres. Figure 2c also shown that the synthesized powder is made from the nano-size particles based on the results of the part particles with shell partially break. The above cleft microspheres probably come from the speedy mass transport pass through the shells. Figure 2d, e also described the structure of Fe_3_O_4_@C. There is a visible core-shell interior structure in Fig. 2d, illuminating that the Fe_3_O_4_ nanoparticles are distributed into the amorphous carbon obviously. This is consistent with SEM result. On the other hand, HRTEM (Fig. 2e) and XRD are also in a good agreement, which show the lattice spacing (0.297 nm) agrees with (220) plane spacing of face-centered cubic Fe_3_O_4_. Its SAED result revealed that the Fe_3_O_4_ nanoparticles were highly crystalline single crystals. Thus, the unique hybrid core-shell sample may hold a higher response efficiency of adsorption site to the target adsorbates, which can be used in the field of environment protection.

**Fig. 3:**
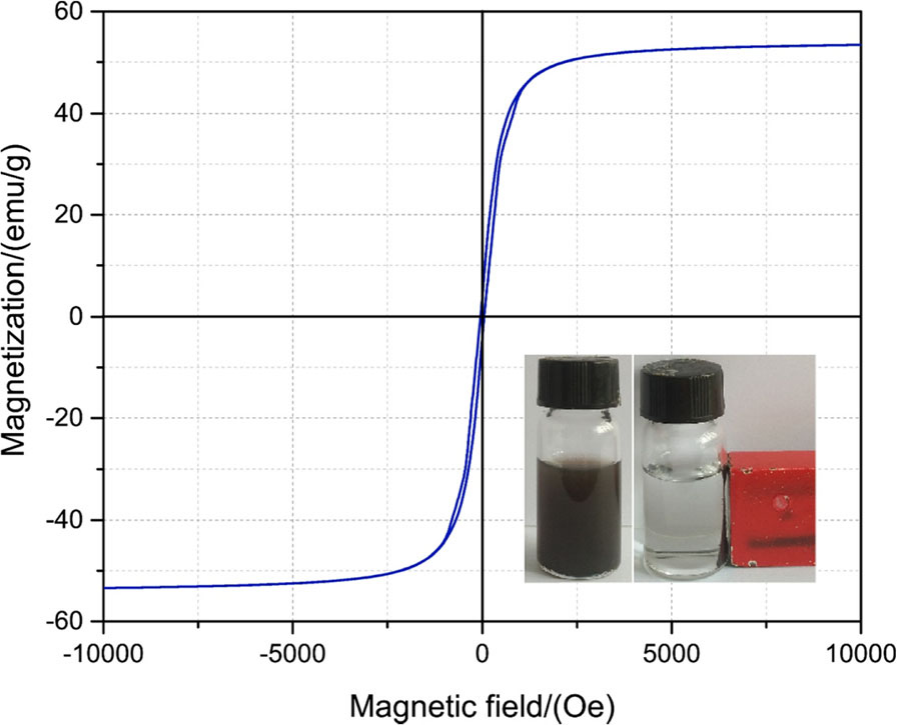
Magnetization loop measurements

The magnetic properties of as-prepared were evaluated by using VSM, and the results are shown in Fig. 3. The magnetic saturation value is reached 53 emu/g compared to bare Fe_3_O_4_ microspheres (67.55 emu/g). The high magnetization value of the prepared material can be clearly seen. The inset in Fig. 3 shows when an external magnetic field was applied, the particles are attracted by the magnet, leaving the aqueous solution clear and transparent.

**Fig. 4:**
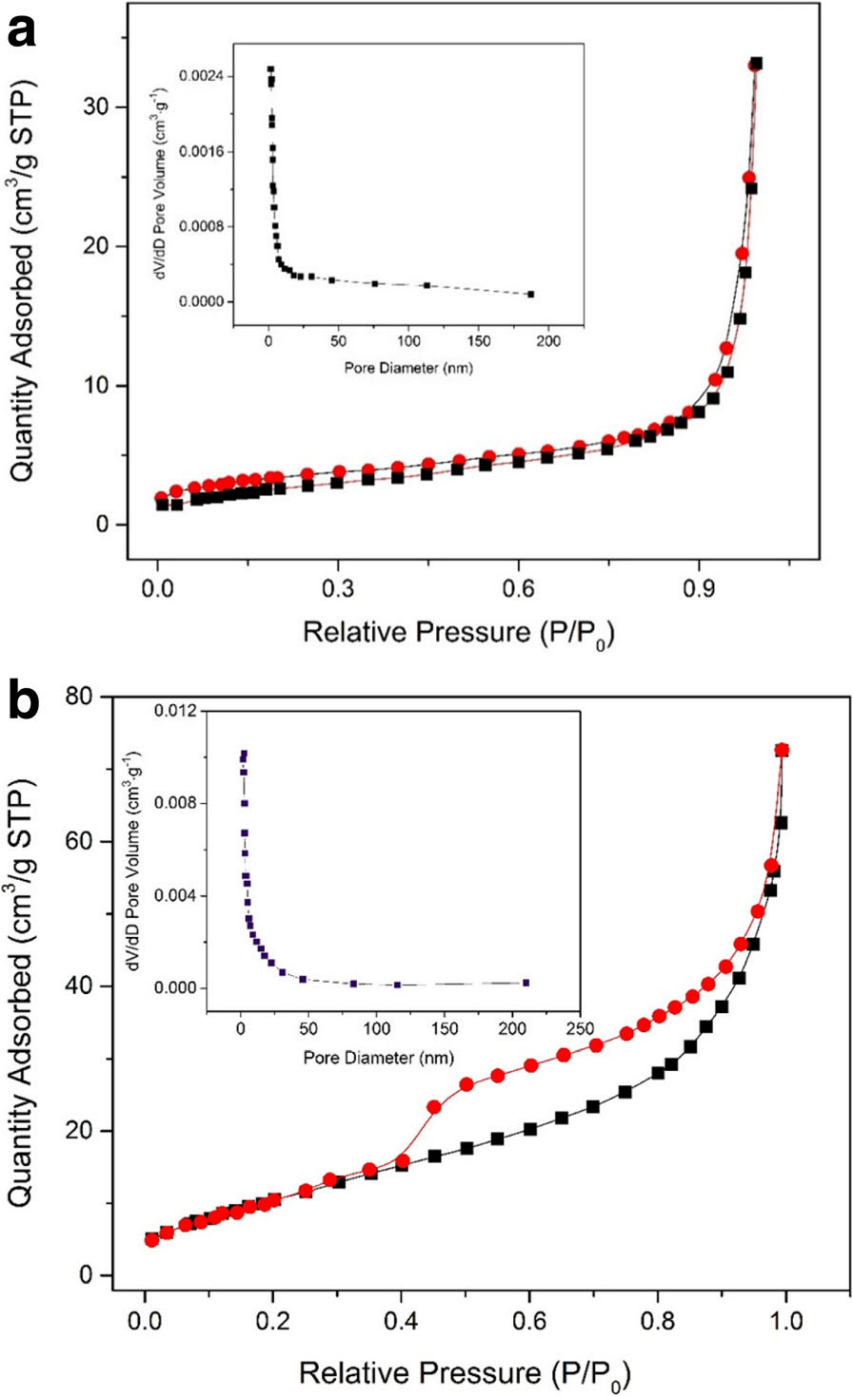
Nitrogen adsorption-desorption isotherm and Barrett-Joyner-Halenda (BJH) pore size distribution plot (inset) of the prepared sample before (**a**) and after (**b**) calcinations

Figure 4a displayed the samples’ adsorption and desorption isotherm, while Fig. 4b shows the pore size distribution of the obtained samples, both of them came from before and after calcinations, respectively. The isotherm is a representative isotherm of mesoporous material based on the classic type IV. The pore size distribution through calcinations clearly shown the average pore diameters from 7.5 to 9.1 nm, smaller than the precursor. Meantime, the sample’s BET surface area increased signally from 9.74 to 238.18 after calcinations, which is higher than most reports [36, 37]. The above also indicated that the abstained Fe_3_O_4_@C samples were a loose mesoporous structure material, which is a benefit for the enhanced adsorption performance.

### Uptake of Heavy Metal Ions by Fe_3_O_4_@C

**Fig. 5:**
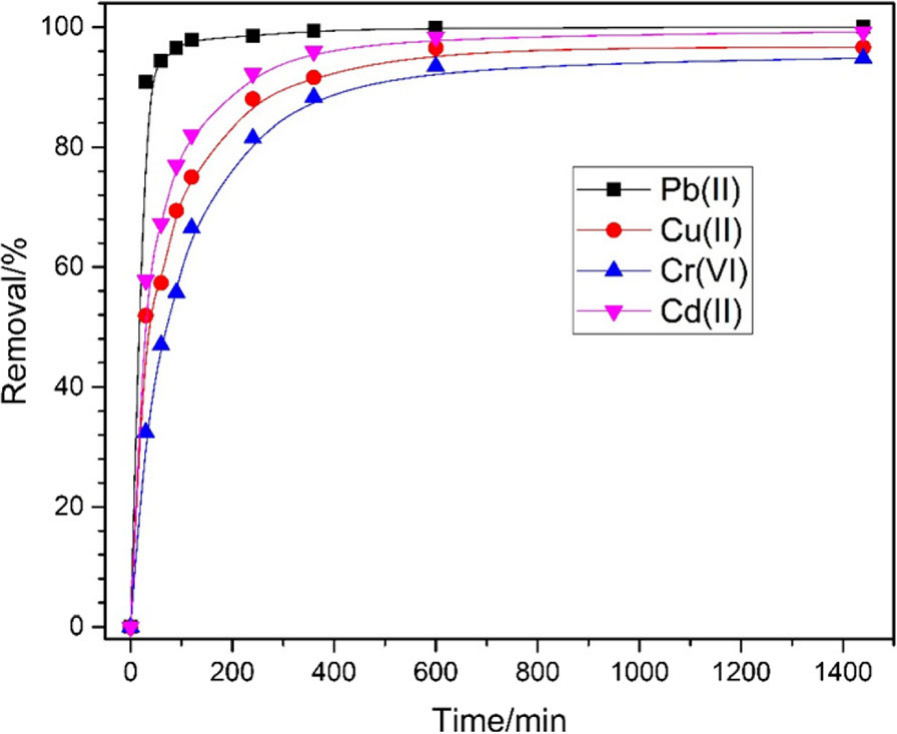
Relationship between the removal efficiency and time for the adsorption of Pb (II), Cd (II), Cu (II), and Cr (VI) by Fe_3_O_4_@C (400 mg L^−1^) sample at initial concentrations of heavy metal ions as 10 mg L^−1^, respectively

Pb (II), Cd (II), Cu (II), and Cr (VI) were chosen as adsorbates for studying the absorption capacity of prepared Fe_3_O_4_@C, and they were put into the pH = 3 at room temperature to conduct kinetic experiments. As shown in Fig. 5, all the adsorption ions successfully adsorbed by the prepared sample, the efficiency reached at 100% for Pb (II), 99.2% for Cd (II), 96.6% for Cu(II), and 94.8% for Cr (VI), respectively. It holds a wider application and higher removal efficiency than the previous study [42]. This high uptake efficiency can be attributed to the intrinsic advantages of core-shell hybrid structures with high specific surface areas, which provided the Fe_3_O_4_@C sample with more active sites for the removal process. Furthermore, it can be easily recovered after adsorption due to the relatively large submicrometer particle structures. It also can be easily perceived that the Pb (II) shown the highest rate which is reasonable due to the adsorption usually by ion-exchange, and there exist electrostatic interaction from the free metal ions and the surface of the adsorbent. So, Cd, Cu, and Cr are light and easy desorptions after ion-exchange, Pb (II) is the opposite [43–49].

### FTIR Spectra of the Heavy Metal Loaded Fe_3_O_4_@C

**Fig. 6:**
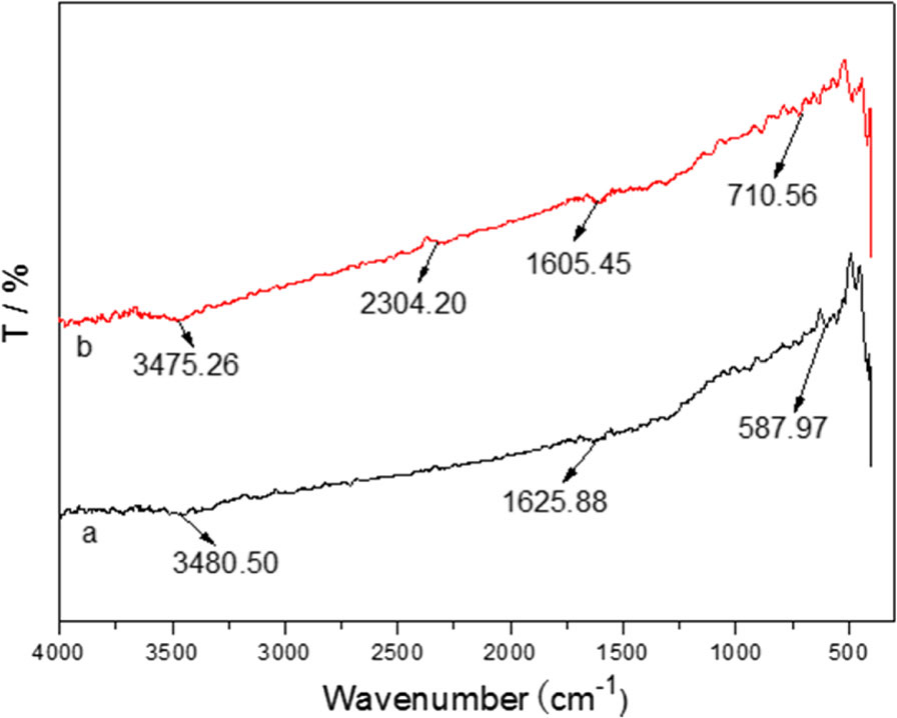
FTIR spectra of the prepared Fe_3_O_4_@C sample before (**a**) and after (**b**) adsorption of Pb (II)

Figure 6 shown the sample with the highest Pb^2+^ uptake at the start and end of the adsorption, which was used in the study for finding out the interaction between Fe_3_O_4_@C and heavy metal ions. Furthermore, the forward and backward vibration of −OH group from H_3_O^+^ via ion exchange or physisorption water molecules, which led to a wide vibrational band at 3475.26 cm^−1^. The peak at 2304.20 cm^−1^ is ascribed to the vibration extend of CO_2_, while the peak at ca. 1625 cm^−1^was usually caused by O-H curve. The peak at 1605.45 cm^−1^ was from carboxyl groups (−C=O−). The main contribution of the other peaks in the range of 400–1000 cm^− 1^ was normally associated with O metal bonds. The difference of the peak position and intensity of metal O in both start and end of the adsorption of Pb^2+^ is implying that Pb^2+^ is loaded on the Fe_3_O_4_@C sample strongly.

### Adsorption Kinetics

**Fig. 7:**
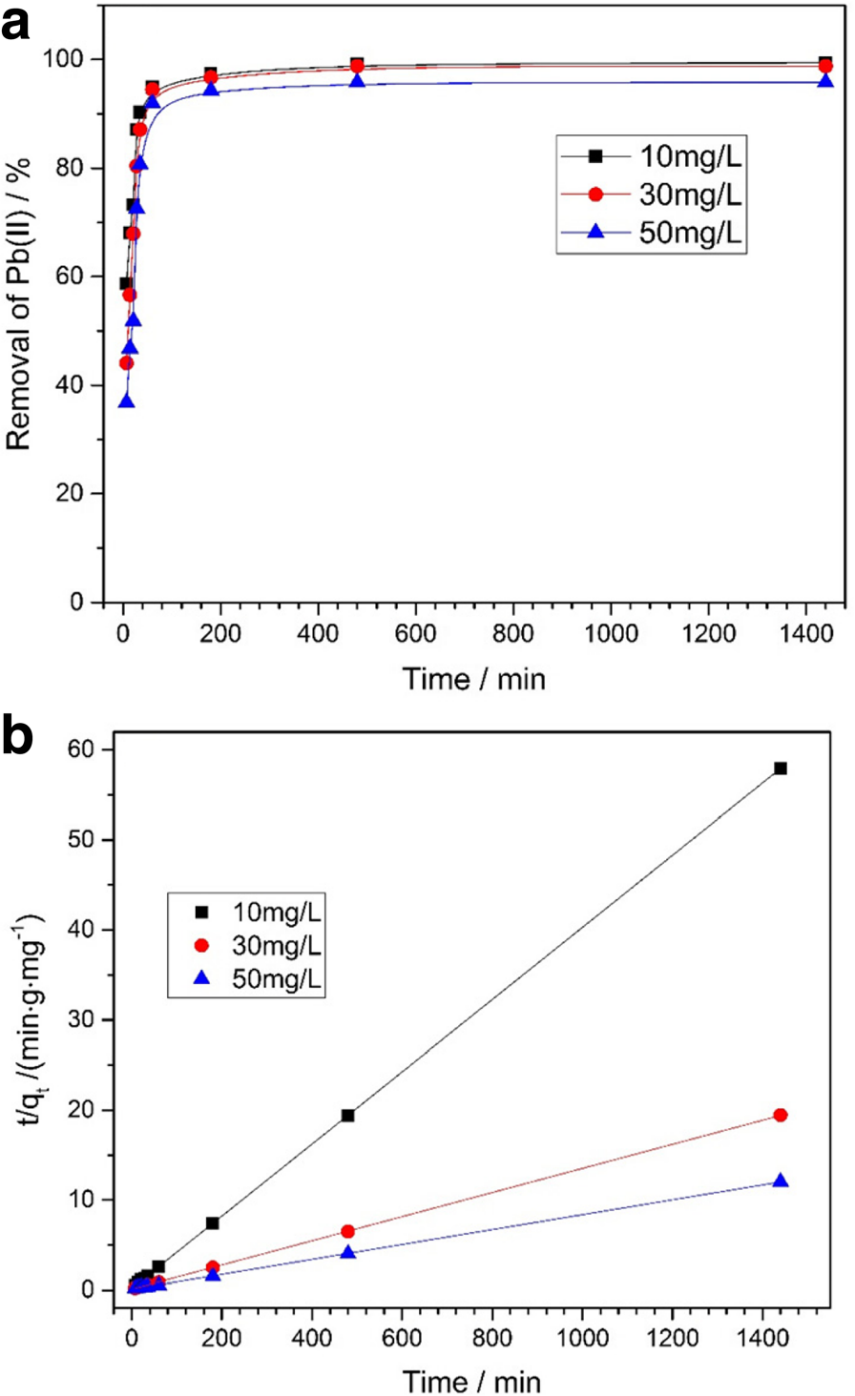
**a** Relationship between the removal efficiency and time for the adsorption of Pb(II) by Fe_3_O_4_@C (20 mg) at initial Pb2+ concentrations of 10, 30, and 50 mg L^−1^, respectively. **b** Pseudo-second-order kinetics for adsorption of Pb2+ on the Fe_3_O_4_@C sample (*T* = 30 °C; absorbent dose = 400 mg L^−1^; Pb2+ concentrations: *a* = 10 mg L^−1^, *b* = 30 mg L^−1^, *c* = 50 mg L^−1^)

A variety of texts were carried out to discuss the adsorption capacity and kinetics of the Fe_3_O_4_@C hybrid nanoparticle aggregates in this section. Pb^2+^ was prepared for removal experiment from the aqueous solutions at pH = 3. After that, prepared the volume of 50 mL of the 10, 30, and 50 mg L^−1^ initial solutions, 20 mg adsorbents, which added to the Pb^2+^ solutions in 100 mL conical flasks in the adsorption of 30 °C respectively. With the different periods (0, 7, 14, 21, 28, 35, 60, 180, 480, and 1440 min), approximately 1 mL was extracted from each solution, they will be used for AAS analysis. Eq. (1) shows the pseudo-second-order kinetic rate model [50]:

1 $$ \frac{t}{q_t}=\frac{1}{k_2{q}_e^2}+\frac{1}{q_e}t $$

where *q*_*e*_ is Pb^2+^ adsorbed per unit mass when the adsorbent is at equilibrium, *q*_*t*_ (mg g^−1^) means the Pb^2+^ adsorbed on unit mass during the time *t* (min); *k*_2_ (g mg^−1^ min^−1^) is the rate constant of the kinetic model, which is a pseudo-second-order. Figure 7a shown the reaction time of the Fe_3_O_4_@C sample in Pb^2+^ removal at the different initial concentrations (10, 30, and 50 mg L^−1^), then the efficiency difference can easily be find out. The result indicated the Fe_3_O_4_@C samples expressed higher adsorption performance, as well separable easily. Figure 7b described the adsorption rate at the different concentrations of Pb^2+^ (10, 30, and 50 mg L^−1^). Adsorption rate was negatively related with the initial concentration. This trend can be revealed as follows, there only had part of the surface-active site is used during the adsorption. Namely, with the concentration of Pb^2+^ increasing, the adsorption started from the high energy sites firstly, followed by the low energy sites saturate that caused the adsorption rate decrease finally [51]. Table 1 shows the correlation coefficient (*R*^2^) reached 0.999 in this study; it indicates clearly the adsorption process conforms well to the pseudo-second-order model.

**Table 1:** The fitted parameters for equilibrium and kinetic adsorption of Pb (II) by Fe_3_O_4_@C sample

Sample	[Pb(II)]_o_ (mg/L)	*q*_*e*,exp_ (mg/g)	*q*_*e*,cal_ (mg/g)	*k*_2_ × 10^−3^ (g mg^−1^ min^−1^)	*R* ^2^	Removal (%, 24 h)
Fe_3_O_4_@C	10	24.84	24.94	8.57	0.999	99.4
	30	74.07	74.51	2.03	0.999	98.7
	50	119.74	120.77	0.89	0.999	95.8

### Adsorption Isotherm

**Fig. 8:**
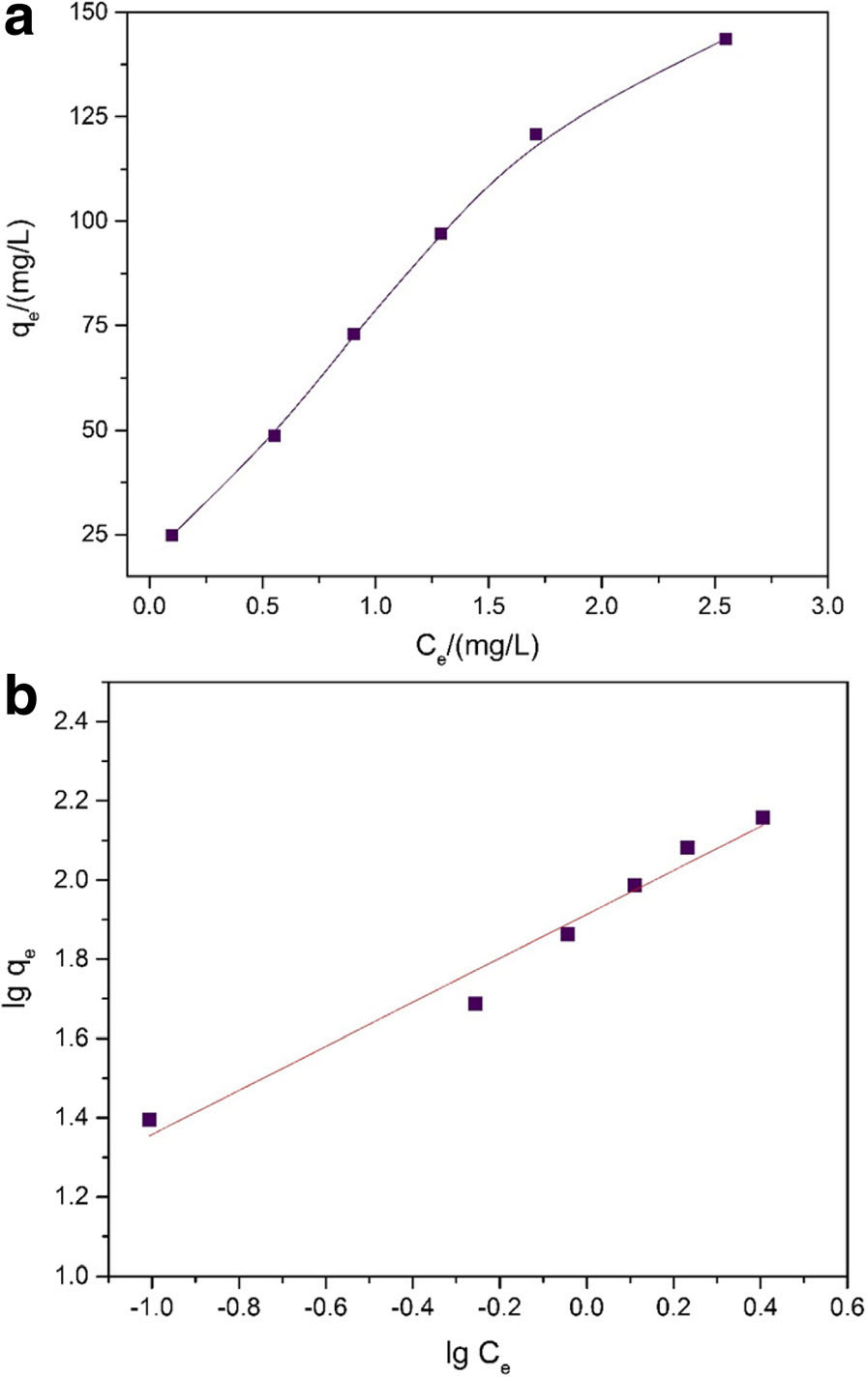
**a** Adsorption isotherm for Pb2+ on the Fe_3_O_4_@C sample (*T* = 30 °C; adsorbent dose = 400 mg L^−1^; Pb2+ concentration = 10–60 mg L^−1^). **b** Freundlich linear plots for adsorption isotherm of the Fe_3_O_4_@Csample on the removal of Pb2+ at 30 °C

In this part, 20 mg adsorbent was added to the 100 mL of conical flasks, and Pb^2+^ solution samples were also prepared (10–60 mg L^−1^, 50 mL, pH = 3). At 30 °C, the above conical flask samples were kept in a sealed condition and placed in a thermostatic shaker (24 h, 150 rpm) and, after that, through centrifugate to obtain the supernatant solution measured by AAS. Figure 8a shown the prepared Fe_3_O_4_@C adsorption ability for Pb^2+^. The Langmuir and Freundlich isotherms were implemented to explain the adsorption course in the study. The process occurred in a localized monolayer, without interaction among adsorbate molecules [52]. In addition, the site for adsorbate molecule is first come first served, no further adsorption in same one site. The Langmuir isotherm described as follow:

2 $$ \frac{C_e}{q_e}=\frac{C_e}{q_{\mathrm{max}}}+\frac{1}{k_L{q}_{\mathrm{max}}} $$

where the theoretical maximum monolayer sorption capacity is represented as *q*_max_ (mg g^−1^), taking *k*_*L*_ to express the Langmuir constant (L mg^−1^), and *C*_*e*_ is the concentration of Pb (II) initially. Whereas, the Langmuir isotherms do not reach the ideal consequence for our study that means it is not well suitable. Corresponding to the linear form as Eq. (3) is another common empirical model as the Freundlich isotherm, which has the hypothesis that, accompany with rising of the site occupation degree, more sturdy binding sites are tied up in advance and its intention decrease correspondingly [52].

3 $$ \lg {q}_e=\lg {k}_F+\frac{1}{n}\lg {C}_e $$

Here, *k*_*F*_ means the Freundlich constant (mg g^−1^)(L mg^−1^)^1/*n*^, and 1/*n* expresses the heterogeneity factor. The specific content of *k*_*F*_ and 1/*n*, identified with a plot of lg *q*_*e*_ versus lg *C*_*e*_, is shown in Fig. 8b. Table 2 shows a favorable adsorption condition [52, 53]. It indicated the reason that the Freundlich exponent *n* is greater than 1. It can be concluded that the adsorption effect of Pb^2+^ was dependent on the hybrid core-shell structures or heterogeneity for the surfaces of the Fe_3_O_4_@C sample. In the meantime, *R*^2^, the correlation coefficient of the sample, reaches up to 0.9712, which signified that Freundlich isotherm model was appropriate for the experimental equilibrium analysis well.

**Table 2:** Freundlich isotherm parameters of the Fe_3_O_4_@C

Sample	*K*_*F*_ (mg g^−1^)(L mg^−1^)^1/*n*^	*n*	*R* ^2^
Fe_3_O_4_@C	81.94	1.80	0.9712

### Thermodynamics Analysis

Considering an isolated system as the relevant assumption, Arrhenius equation (Eq. (4)) was taken to conduct the thermodynamics analysis. Typically, under some special conditions of thermostatic shaker that original concentration was 30 mg L^−1^ and the adsorption volume was 50 ml, then, 20 mg adsorbent was put into the conical flasks with capacity of 100 mL as well as Pb^2+^ solutions at five classification temperature conditions including 30, 40, 50, 60, and 70 °C. In the process of adsorption, the aqueous samples were conducted sampling after various time buckets; during this period, the consistent concentrations of Pb^2+^ were also to be measured based on the AAS which was used to analyse its adsorption kinetics according to the above different temperature situation. Besides, the Arrhenius equation given in the previous is utilized to evaluate the activation energy which was the precondition for the adsorption research.

4 $$ \ln {k}_2=1n\kern0.5em A-\frac{E_a}{RT} $$

**Fig. 9:**
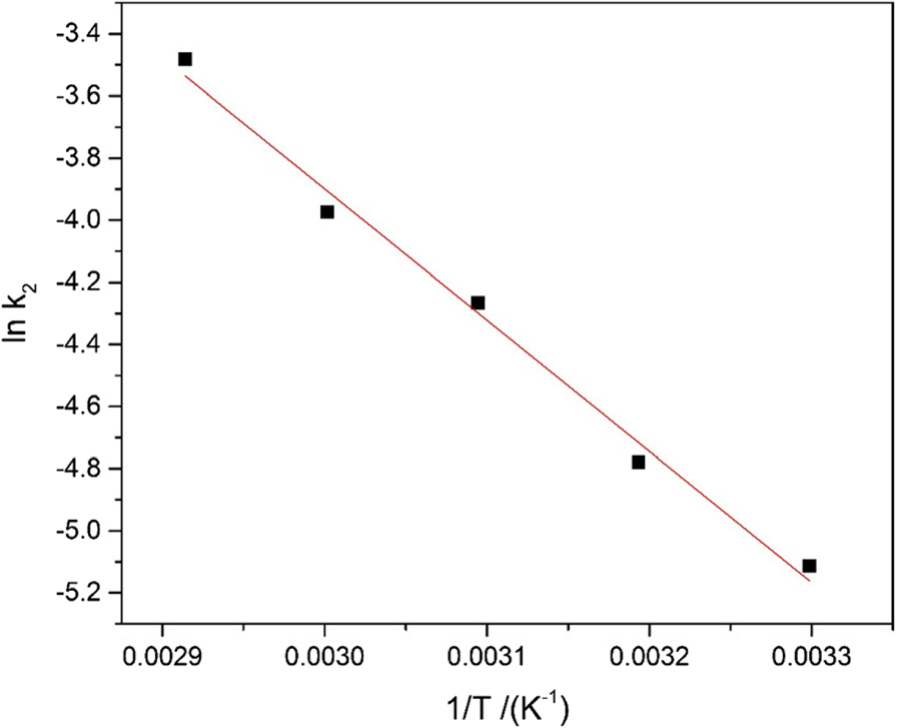
Plot of lnk2versus 1/*T* for Pb2+ adsorption on the Fe_3_O_4_@C sample (TOC)

Here, *E*_*a*_ is the Arrhenius activation energy (kJ mol^−1^). *A* represents the factor of Arrhenius; the gas constant (8.314 J mol^−1^ K^−1^) is expressed with *R*, and *T* refers to the absolute temperature (K). The corresponding slope is −*Ea*/*R*, based on a plot of ln*k*_2_ against 1/*T* (Fig. 9) to get the straight line.

The dimensions of activation energy were taken to determine the form of adsorption. Usually having a specific scope (0–40 kJ mol^−1^) for the activation energy in the process of physisorption [54], a longer range was needed in chemisorption process by contrast. The activation energy was 34.92 kJ mol^−1^ here. It indicated that the adsorption process of Pb^2+^ onto the Fe_3_O_4_@C is classified into physisorption.

## Conclusions

The compound of core-shelled Fe_3_O_4_@C hybrid nanoparticle aggregates is achieved through adopting pacific and moderate steps environmentally based on the solvothermal synthesis method and obtained the calcinations ultimately at 450 °C. Via the carbon-based hybrid core-shell nanostructures, a greater degree of exposure efficiency of adsorption sites can be realized efficiently for the adsorbate when compared to a solid one, which will deliver adsorption properties better to eliminate the heavy metal ions. Additionally, the iron-based cores make the adsorbents be separated easily from the aqueous solutions. Under this device (cheaper, less complicity, and higher productivity), a new approach is clarified that core-shell nano/micro-functional materials can be synthesized well on a large scale which are used in many fields such as environmental remediation, catalyst, and energy.

## Abbreviations

AAS: Atomic absorption spectroscopy
BET: Brunauer-Emmet-Teller
FTIR: Fourier transform infrared spectroscopy
HR-TEM: High-resolution transmission electron microscope
JCPDS: Joint Committee on Powder Diffraction Standards
SAED: Selected area electron diffraction
SEM: Scanning electron microscope
TEM: Transmission electron microscope
VSM: Vibrating-sample magnetometer
XRD: X-ray diffraction
